# ePlant and the 3D Data Display Initiative: Integrative Systems Biology on the World Wide Web

**DOI:** 10.1371/journal.pone.0015237

**Published:** 2011-01-10

**Authors:** Geoffrey Fucile, David Di Biase, Hardeep Nahal, Garon La, Shokoufeh Khodabandeh, Yani Chen, Kante Easley, Dinesh Christendat, Lawrence Kelley, Nicholas J. Provart

**Affiliations:** 1 Department of Cell and Systems Biology, University of Toronto, Toronto, Canada; 2 Centre for the Analysis of Genome Evolution and Function, University of Toronto, Toronto, Canada; 3 Department of Computer Science, University of Toronto, Toronto, Canada; 4 Structural Bioinformatics Group, Division of Molecular Biosciences, Department of Life Sciences, Imperial College London, London, United Kingdom; Michigan State University, United States of America

## Abstract

Visualization tools for biological data are often limited in their ability to interactively integrate data at multiple scales. These computational tools are also typically limited by two-dimensional displays and programmatic implementations that require separate configurations for each of the user's computing devices and recompilation for functional expansion. Towards overcoming these limitations we have developed “ePlant” (http://bar.utoronto.ca/eplant) – a suite of open-source world wide web-based tools for the visualization of large-scale data sets from the model organism *Arabidopsis thaliana*. These tools display data spanning multiple biological scales on interactive three-dimensional models. Currently, ePlant consists of the following modules: a sequence conservation explorer that includes homology relationships and single nucleotide polymorphism data, a protein structure model explorer, a molecular interaction network explorer, a gene product subcellular localization explorer, and a gene expression pattern explorer. The ePlant's protein structure explorer module represents experimentally determined and theoretical structures covering >70% of the Arabidopsis proteome. The ePlant framework is accessed entirely through a web browser, and is therefore platform-independent. It can be applied to any model organism. To facilitate the development of three-dimensional displays of biological data on the world wide web we have established the “3D Data Display Initiative” (http://3ddi.org).

## Introduction

Model organisms are essential research tools for the biological sciences. Harnessing the full power of these model systems requires integrating data from the many diverse scales of their environment and physiology, and intuitive data displays that are accessible, comprehensible, and expandable by researchers from a broad range of disciplines [1]–[7]. For the model plant *Arabidopsis thaliana*, large-scale data sets have been generated at many different biological scales [8] and several algorithms are available to identify associations among these levels [9], [10] towards a systems understanding of biological processes. However, the tools available to interpret these immense data sets are limited with respect to visualization, accessibility and expansion, and the scope of data integration. The integrated and interactive visualization of a broad range of data types has been identified as a fundamental challenge for the future of systems biology [11], [12]. Effective data visualization facilitates the discovery of relationships between diverse data sets [13] and is therefore critical for integrative systems biology. With the exception of molecular structure viewers, the visualization of biological data has largely been trapped in two-dimensional (2D) representations. 2D data displays are limiting in two important aspects – humans have evolved an exceptional ability to visualize and conceptualize in the three spatial dimensions of our universe, and displaying these dimensions is critical to all fields of biology. Consider the relationship between protein fold and function, cellular polarity and development, the geographic distribution of organisms and evolution, and the interactions between each of these scopes. Three-dimensional (3D) data displays thus represent enormous potential for the biological sciences, particularly with consideration for the coming widespread availability of display technologies capable of creating 3D images autostereoscopically without requiring special eyewear for users to perceive them [14]. The utility of mapping biological omics data onto 3D anatomical reconstructions of model organisms has been demonstrated in the mouse brain [15].

Although many excellent computational systems biology tools have been developed with publicly available source code, they are typically encapsulated in one programmatic language without modular compatibility to other programs and must be recompiled for functional expansion [16]–[21]. The accessibility of systems biology data is further limited by the frequent requirement for biologists to download, install, and configure data visualization and analysis software for their specific operating system. The status quo can thus impose steep learning curves and other barriers to user community-driven expansion of systems biology software. These limitations can be addressed through software development on the world wide web [22]. Data accessibility and maintenance can be greatly improved through web services [23], and data display frameworks designed for web browsers and common scripting languages maximize their accessibility and facilitate their functional expansion by user communities (for example, Jmol: [24]). Recent years have seen remarkable developments in online resources for molecular systems biology [25]. For example, BioCyc [26], Reactome [27]–[29], and KEGG [30] are vast repositories of manually curated and publicly accessible molecular biological data. These tools allow some degree of data integration. However these tools are represented in 2D and are either network- or pathway-centric and are limited in their range of integrated biological scales.

Herein we report an open-source template for the integration and visualization of systems biology data as interactive 3D representations on the world wide web. We have applied this framework to the important model plant *Arabidopsis thaliana* in the form of “ePlant” (http://bar.utoronto.ca/eplant). To take advantage of the ePlant framework we generated a proteome-scale protein structure prediction and annotation for Arabidopsis and integrated existing omics-scale data for Arabidopsis. The template used to construct ePlant can be applied to any model organism to achieve intuitive and efficient data retrieval and display. To facilitate the development of 3D data display on the world wide web we have also established the “3D Data Display Initiative” (3DDI - http://3ddi.org). The ePlant framework can be flexibly modified and interact with other web services and data display modules. With only an identifier for a gene of interest, ePlant users can rapidly evaluate protein structure and function, protein-protein interactions, protein subcellular localization, gene expression patterns, and genetic variation. This integrates biological data from nanometer-scale molecular processes to genetic variation based on kilometer-scale geographic distributions. ePlant users can contemplate the relationships between these properties and their genes of interest towards a systems level understanding of model organism biology.

## Results and Discussion

### Querying the ePlant Data Display Modules

Gene products, such as proteins and RNA transcripts, and many important physiological phenotypes can be unambiguously linked to gene identifiers. An ePlant query thus begins with entering an Arabidopsis Genome Initiative gene identifier (AGI-GI) on the main query page and selecting one of the available modules to explore the properties associated with a query gene and its products. Biological data for the model organism *Arabidopsis thaliana* is rendered as an interactive 3D display module within the web browser. Currently, ePlant consists of the following modules: a sequence conservation explorer, a protein structure model explorer, a molecular interaction network explorer, a gene product sub-cellular localization explorer, and a gene expression pattern explorer. This form of semantic zooming facilitates the integration of biological data across several scales.

### Proteome-Wide Protein Structure Prediction for the Model Plant Arabidopsis

**Figure 1:**
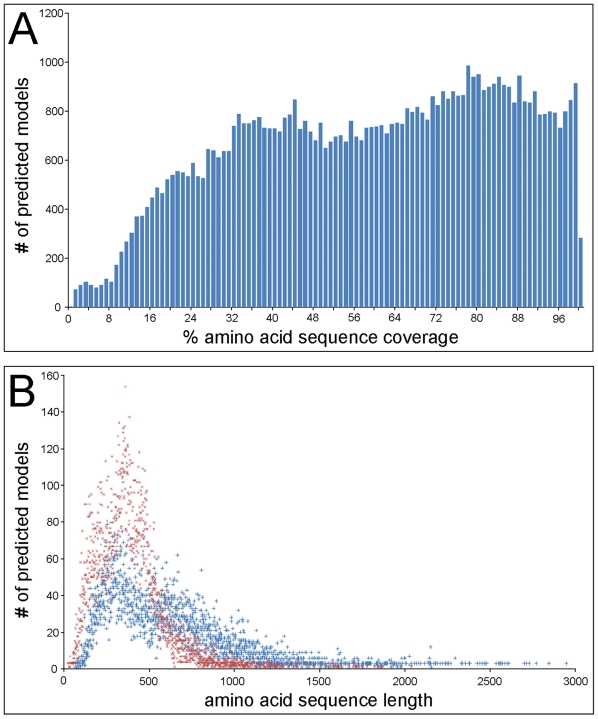
Sequence coverage performance for the Arabidopsis whole-proteome protein structure prediction. A) A bar graph showing the number of Phyre-predicted [32] protein structure models from the TAIR9 Arabidopsis proteome by percent amino acid sequence coverage of the model based on the full-length TAIR9 template sequence. B) A scatter plot of the number of predicted protein models versus amino acid sequence length of the models for those models with percent sequence coverage between 0–55% (X) and 56–100% (+).

**Figure 2:**
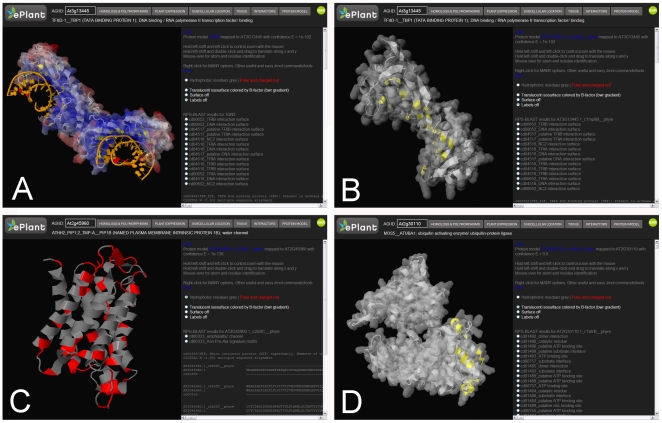
The ePlant Protein Structure Model Explorer. A) Screenshot depicting the x-ray crystallographic dimeric protein structure model of Arabidopsis TATA binding protein 1 (TBP1, At3g13445) complexed with DNA, PDB:1QN3. The Jmol [24] van der Waal's surface rendering of the protein is colored with a blue to red gradient representing low to high mobility as calculated by the temperature factor of the model. The alpha carbons of the sites defining the DNA interaction surface as curated by the Conserved Domain [33]–[34] entry CD00652 are highlighted in yellow. B) Screenshot depicting the predicted monomeric protein structure model of Arabidopsis TBP1 (At3g13445), built with PDB:1MP9 as a template for homology modeling using Phyre [32]. The alpha carbons of the sites defining the DNA interaction surface as curated by the Conserved Domain entry CD00652 are highlighted in yellow. The Jmol van der Waal's surface rendering is monochromatic grey as temperature factors are not currently calculated for predicted structures. C) Screenshot depicting the predicted protein structure model for the Arabidopsis water channel protein encoded by At2g45960. The ribbon model is colored grey for hydrophobic residues, which reside in the plasma membrane, and colored red for polar and charged residues which face the aqueous cellular interior and solvent exterior. D) Screenshot depicting the predicted protein structure model for the Arabidopsis ubiquitin-protein ligase encoded by At2g30110. The protein is shown as a ribbon diagram with a monochromatic grey surface. The loop and helical regions colored in yellow define the heterodimer interaction surface of the protein, described by CD01493.

The 3D structure of proteins can provide a wealth of information regarding their biological functions [31]. However, while there are ∼34,000 polypeptides in the most recent TAIR9 collection of Arabidopsis proteins (http://www.arabidopsis.org) the Protein Data Bank (http://www.rcsb.org) contains only ∼62,000 macromolecular structures, with 2488 structure models from the *Viridiplantae* and only 495 from Arabidopsis at the time of preparing this manuscript. It is therefore difficult for researchers to find protein structural data directly related to their genes of interest. To address this knowledge gap we determined theoretical protein structures for the Arabidopsis proteome using the Phyre homology modeling method [32] with the TAIR9 proteome, including splice variants, as input sequences. We obtained 67,275 predicted protein structure models with the highest level of confidence, as per [32], for ∼72% of the Arabidopsis proteome. Most of the predicted protein structures span less than the entire amino acid sequence of each TAIR9 polypeptide. This results from the current implementation of Phyre which uses one template for homology modeling per protein sequence. The distribution of percent amino acid sequence coverage for this collection of predicted protein structures is bi-modal, with one peak at ∼35% coverage and the other at ∼80% coverage (Figure 1A). The distribution of sequence length in these two modes reveals that Phyre typically achieves greater sequence coverage for longer protein sequences (Figure 1B). The percent sequence coverage reported in Figure 1 understates the total sequence coverage for a given TAIR9 protein, as up to three predicted protein structures were generated for each TAIR9 protein sequence and in some cases map to separate sequence regions. Forthcoming implementations of Phyre can integrate multiple independent structure prediction templates to produce one multi-domain protein structure model. The Phyre models and the mapping of curated sites onto these models can be validated against experimentally characterized protein structures (Figure 2A–2B and Figure S1).

### ePlant Protein Structure Explorer

The amount of functional data that can be extracted from 3D protein structures will be enhanced with readily accessible visualization and annotation tools. Careful comparative analysis and annotation of key features of protein structure are critical to linking protein fold with biological function. We have implemented a novel protein structure annotation scheme through the Javascript interface to the Jmol rendering engine. Specifically, we integrate biological and protein structural data by mapping annotated domains and amino acids of curated functional importance [33], [34] onto the 3D protein model. For example, Figure 2A illustrates the DNA interaction surface mapped onto the Arabidopsis DNA binding protein TBP1, encoded by At3g13445 and represented by the crystallographic protein structure model PDB:1QN3. Figure 2B shows the same DNA interaction surface mapped onto a predicted protein structure for TBP1 (At3g13445), modeled using PDB:1MP9 as a template. The mapping of curated sites is consistent between the experimental (Figure 2A) and predicted (Figure 2B) protein structure model for TBP1 (At3g13445). Curated sites mapped onto a structure are highlighted by selecting radio buttons. Multiple sequence alignments and text-based annotations associated with these curated sites are also displayed in the ePlant Protein Structure Model Explorer to provide context and validation of these site mappings. Mapping conserved sites of functional importance in their 3D context is more informative than lists or 2D schematics (Figure S2) of conserved sites as these often cluster spatially in 3D as sectors to define functional surfaces and other functionally important structures despite being distributed throughout the linear primary sequence. The side-chain orientations of predicted protein structures are not accurate, therefore the default display is a cartoon representation of secondary structure elements. We expect that in most cases the surfaces defined by mapped CDD sites, such as the DNA-binding region of At3g13445 shown in Figure 2A,B and the protein-protein interaction interface shown in Figure 2D, are more informative and accurate than the specific properties and orientations of the mapped amino acids. All of the Phyre predicted protein structure models for Arabidopsis, and all of the experimentally determined Arabidopsis protein structures at the Protein Data Bank, are accessible for download or viewing in the ePlant Protein Structure Explorer. This module is powered by the Jmol web applet [24], which provides a scripting interface and a wealth of features for analyzing molecular structures. Useful functions for protein structure analysis such as molecular surfaces with scaled color gradient displays based on metrics stored in the mobility/temperature factor field of the PDB files (Figure 2A) or colored mappings of hydrophobicity, polarity and charge states on ribbon diagrams (Figure 2C) have been pre-computed to be easily accessed through radio buttons in the ePlant Protein Structure Model Explorer.

### ePlant Sequence Explorer

**Figure 3:**
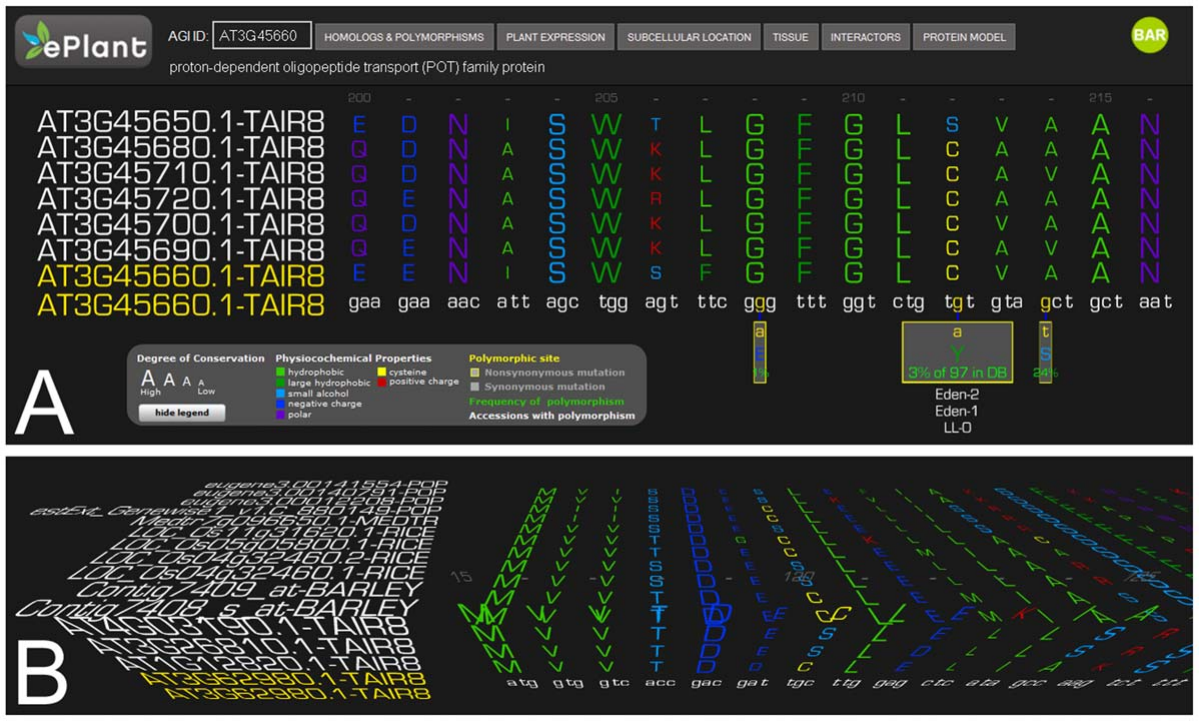
The ePlant Sequence Explorer. A) Screenshot depicting a cluster of polymorphic sites mapped to the locus At3g45660. B) Screenshot depicting a 3D alignment of putative orthologs and paralogs of TIR1 auxin receptor encoded by At3g62980.

The ability to assess sequence conservation within and between species is informative regarding sequence evolution and is critical to understanding the function of gene products. When integrated with knowledge of the geographical distribution of species and their genetic variation, these data provide kilometer scale resolution for the biology of model organisms. The ePlant sequence explorer is a first attempt at the 3D display of primary sequence data. We have incorporated 123,484 single nucleotide polymorphisms using data from [35], [36] into the ePlant Sequence Explorer. Figure 3A shows a cluster of polymorphic sites for an oxidoreductase encoded by At4g04930. The interactive display indicates synonymous and non-synonymous polymorphic sites, the alternate amino acid encoded by non-synonymous polymorphisms, and the frequency and ecotypic distribution at each polymorphic site. Figure 3B illustrates the 3D representation of sequence data for the TIR1 auxin receptor encoded by At3g62980. Amino acid sequences of paralogs and putative orthologs (computed by Patel R and Provart NJ et al., manuscript in preparation) of the query gene are aligned across separate axes, with putative orthologous sequences at a plane that is orthogonal to the paralogs and splice variants of the query Arabidopsis gene. For example, this 3D multiple sequence alignment may be scrolled and rotated to view only the putatative orthologs or paralogs. Each of the one letter amino acid sites in the ePlant Structure Explorer are rendered as separate objects to integrate additional biological data. The color of the one-letter amino acid codes represents physico-chemical properties such as charge and solubility and the size of the letters are scaled to conservation scores in the alignment. This also allows the integration of primary sequence data with the cognate folded 3D protein structure by means of hyperlinks. Each letter is clickable, allowing the user to see the location of the residue in the cognate structure in the ePlant protein structure model explorer. Upon clicking a residue of interest, the user is prompted to select a protein structure related to their query sequence, which is rendered in the structure explorer module with the residue of interest labeled and highlighted in red.

### ePlant Molecular Interactions Explorer

**Figure 4:**
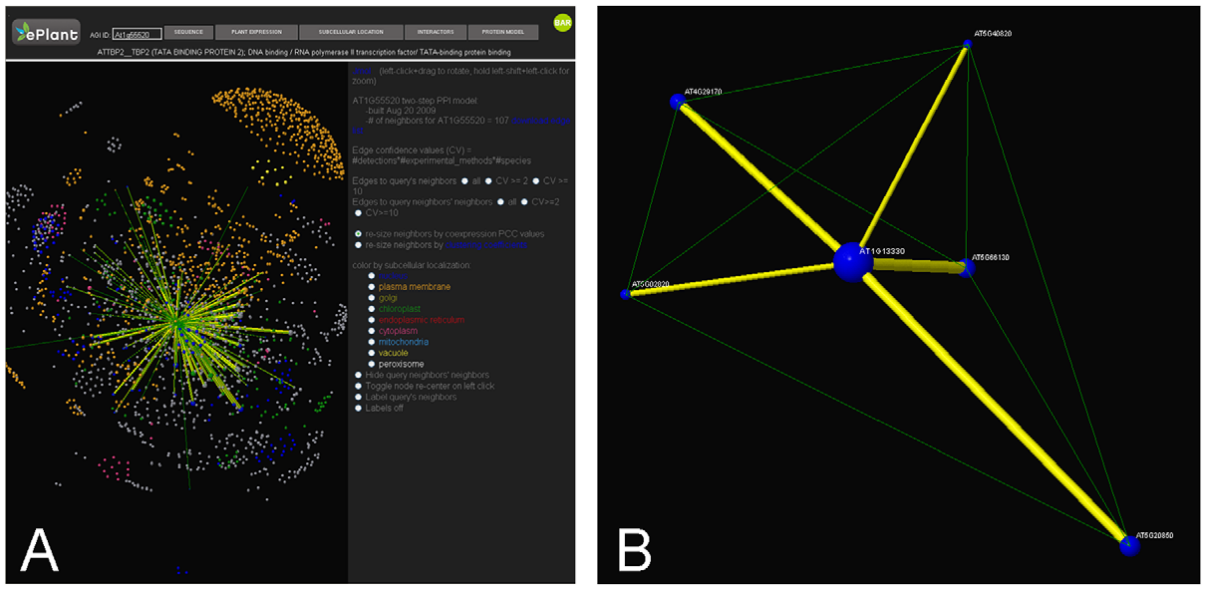
The ePlant Molecular Interactions Viewer. A) Screenshot displaying the “two-step” protein-protein interaction network centered on the Arabidopsis TBP1 (At3g13445). B) Protein-protein interaction network centered on an Arabidopsis protein of unknown function encoded by At1g13330. This layout was created by first re-sizing the query neighbors by mRNA expression correlation coefficients (shown in yellow), followed by selecting all edges between the query neighbors' neighbors and hiding the query neighbors' neighbors. As a result, the green edges depict interactions between the query's neighbors and can be used to validate network clustering coefficients calculations. The query node is scaled by its clustering coefficient (0.80), and all nodes are colored blue based on nuclear localization.

The abstraction of biological systems as networks is a powerful approach to understanding their functions [37], [38]. Representing biological networks in 3D can facilitate user interaction with large and complicated data sets [21]. The ePlant Molecular Interactions Viewer is a template for the visualization and analysis of biological networks in 3D using the Jmol rendering engine [24] and therefore has the advantage of deployment on the web and functional expansion through scripting. This module currently supports the interactive exploration of 70,944 predicted and 4,300 documented Arabidopsis protein-protein interactions, derived from [39], [40] and others. Proteins are represented as spheres and edges connecting the spheres indicate undirected protein-protein interactions. The models are centered on the protein product of the query gene and by default display edges to the query's interaction neighbors as well as the query neighbors' neighbors. These “two-step” models thus describe the local protein-protein interaction neighborhood of the query gene. The large two-step interaction network centered on Arabidopsis TBP1, encoded by At3g13445, is shown in Figure 4A. Nodes and edges can be rendered dynamically to reflect additional data sets and network properties. For example, nodes can be colored to indicate sub-cellular localizations of the proteins in the two-step network, the size of nodes and edges can be rendered to reflect metrics of interaction confidence values, the expression correlation of the mRNA transcripts associated with the proteins in the two-step network, or other network properties such as clustering coefficients (Figure 4B). Protein interaction partners and other network data are available for download as plain text from the ePlant Molecular Interactions Viewer. Network topologies in the two-step protein-protein interaction models can illustrate ordered structures related to underlying biological phenomena such as protein complex formation and the connectivity between functional modules such as signal transduction pathways between sub-cellular compartments [37], [38]. As networks are useful abstractions for many systems outside the realm of biology, our method for rendering networks in 3D on the web may find application to other fields of inquiry.

### Gene Expression Patterns and Gene Product Localization at Tissue and Subcellular Scales

**Figure 5:**
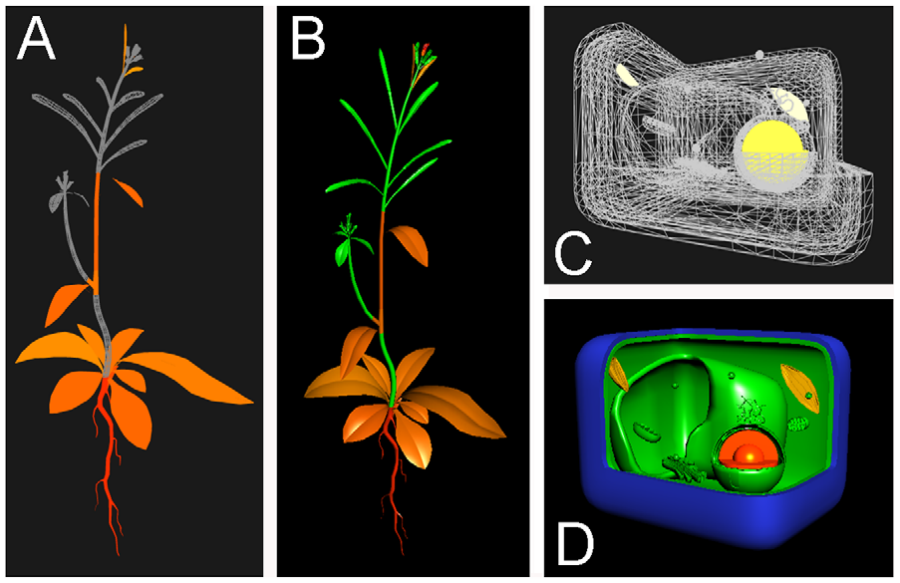
The ePlant Expression Pattern and Subcellular Localization Explorers. A) Screenshot showing the gene expression patterns for Arabidopsis TBP1 (At3g13445) using the PaperVision3D rendering engine. B) O3D rendering of the the same model of gene expression for Arabidopsis TBP1 (At3g13445) as shown in panel A. C) ePlant Subcellular Localization Explorer showing the nuclear localization signal for Arabidopsis TBP1 (At3g13445) in a wireframe cartoon representation of a plant cell, rendered with PaperVision3D. D) O3D rendering of the same model of subcellular localization for Arabidopsis TBP1 (At3g13445) shown in panel C.

**Figure 6:**
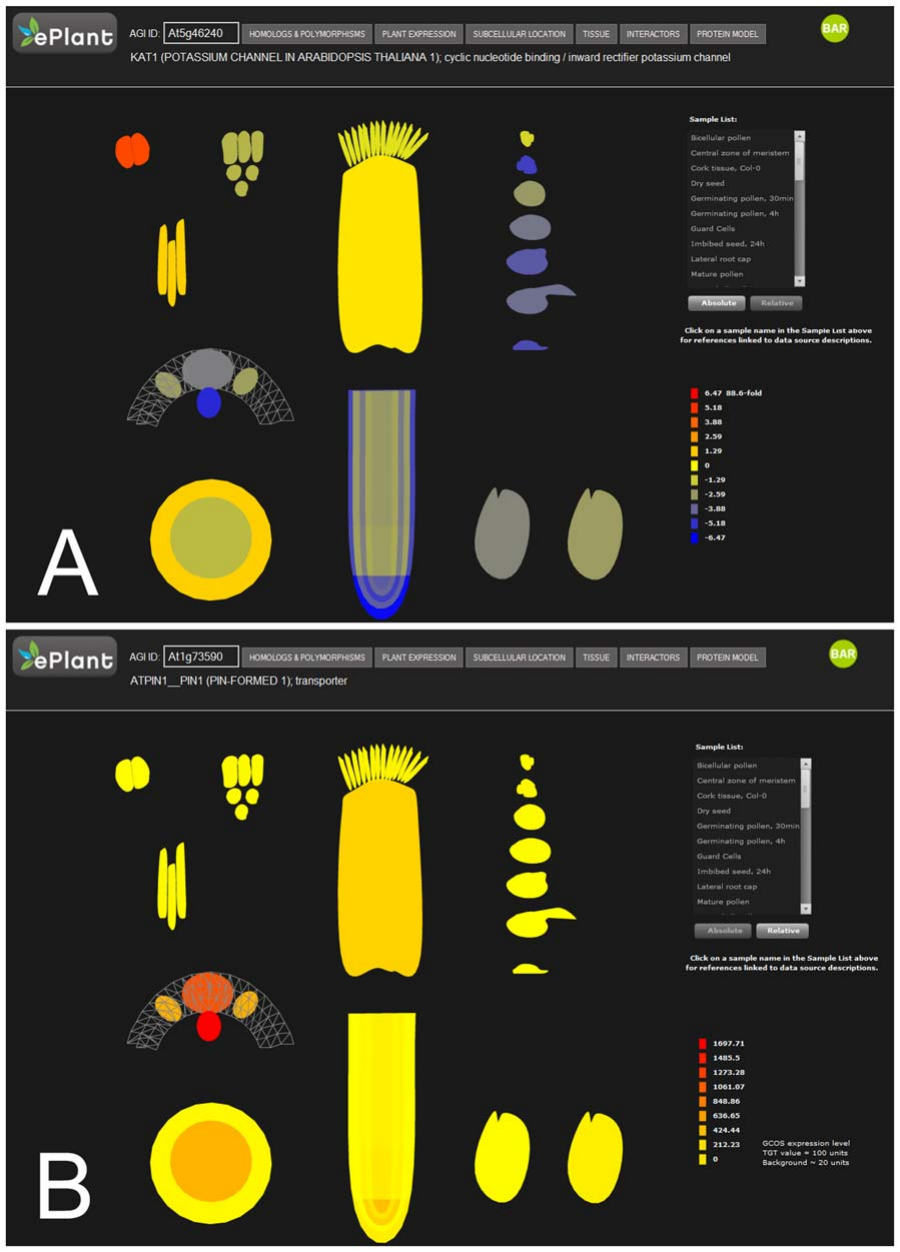
The ePlant Tissue Expression Explorer. A) Screenshot showing the relative gene expression level of the potassium transporter encoded by At5g46140 across several tissue types. B) Screenshot showing the absolute gene expression level of the auxin transporter encoded by At1g73590. Individual tissue types can be identified by name and source data by clicking the identifiers in the list.

We have developed gross anatomical, tissue level, and sub-cellular models of *Arabidopsis thaliana* to integrate molecular omics data at physiological scales (mm-cm). For example, the mRNA expression patterns of a gene can be painted onto a 3D anatomical model of Arabidopsis (Figure 5A,B) and the subcellular localization of a gene's protein product can also be painted onto a 3D model of a plant cell (Figure 5C,D). Gene expression data for the whole-plant model of Arabidopsis are from [41]. Subcellular gene product localization data are from the SUBA database [42]. At the ‘Tissue Expression’ level, expression data from 34 different tissues and cell types may be explored (Figure 6A,B). The data are from guard cells and mesophyll cells [43] (http://biology.ucsd.edu/labs/schroeder/guardcellchips.html), xylem and cork (Campbell M, unpublished - http://affymetrix.arabidopsis.info/narrays/experimentpage.pl?experimentid=92), stigma and ovaries [44], stem epidermis [45], 15 cell types from 5 layers and 3 ages of the root [46], three areas of the shoot apical meristem [47], four pollen developmental stages [48], dry and imbibed seeds [49], and three pollen germination stages [50]. ePlant permits easy access to 2.78 million gene expression measurements, and documented subcellular localizations for 6,897 Arabidopsis proteins and predicted subcellular localizations for most of the remainder of the Arabidopsis proteome. The Arabidopsis whole-plant, tissue, and cellular models are specified by the Collada Data Asset Exchange format (http://collada.org) and rendered in the web browser using PaperVision3D (http://papervision3D.org) (Figure 5A,C) or Google's O3D (http://code.google.com/apis/o3d) (Figure 5B,D). A prototype using the latter is available at http://3DDI.org. These models can be freely rotated and signal values can be painted dynamically.

## Materials and Methods

### Data Storage and Retrieval

The fast performance of ePlant is achieved by rapid data transfer via JSON-type Representational State Transfer (REST) web services, server-side MySQL database queries at the Bio-Array Resource (http://bar.utoronto.ca) or the SUBA database [42] (http://suba.plantenergy.uwa.edu.au), and the ability of web browser-based rendering engines to use the memory and processing resources on the ePlant user's local computing device. All sequence data for Arabidopsis was retrieved from The Arabidopsis Information Resource (http://www.arabidopsis.org). Homologous sequences were retrieved from the National Center for Biotechnology Information (http://ncbi.nlm.nih.gov) or TIGR (Patel R and Provart NJ et al., manuscript in preparation). Protein sub-cellular localization assignments are retrieved via webservices from SUBA [42].

### Arabidopsis Protein Structure Prediction and Annotation

The TAIR9 protein sequences, including splice variants, were used as input for the high-throughput structure prediction of the Arabidopsis proteome. Phyre models of the TAIR9 protein sequences with confidence values of 100% as per [32] were considered significant. TAIR9 protein sequences were mapped to the implicit protein sequence from each of the structure model PDB files using BLAST-P with an expect value E< 1e-5. Structure model implicit sequences were then compared to the Conserved Domain Database (CDD) [33], [34] using RPS-BLAST. Matches to CDD profiles with E< 1e-5 were considered significant. Curated CDD amino acid sites of functional importance were mapped from cddannot.dat and the CDD master sequence onto the implicit primary sequence of the protein structure model sequence using Bioperl [51] methods on the RPS-BLAST alignments. Briefly, the site mapping algorithm counts the gaps in the homology string sequence for each RPS-BLAST hit to map the curated sites in the CDD master sequence onto the implicit PDB sequence. Multiple sequence alignment displays in the ePlant Molecular Interactions Explorer of the implicit protein model, CDD profile, and query sequences are generated on the server-side with ClustalW [52] using a BLOSUM matrix.

### Arabidopsis Protein-Protein Interaction Network Layout

The 3D layout of the two-step networks were computed by the Mathematica 7.0 kernel (64-bit) [53] on a Linux architecture using the ‘GraphCoordinates3D’ function with the ‘Method’ parameter set to ‘Automatic’. The coordinates of each node in the 3D layout were retrieved using the Mathematica function ‘VertexList’ and the ‘GraphUtilities’ library [53]. Perl scripts were used to pass the network edges from a MySQL database on the BAR server to Mathematica and convert the 3D coordinates of the nodes in the two-step network to the .XYZ format for rendering in the Jmol applet [24]. There is a separate .xyz layout file for each TAIR9 protein with one or more documented or predicted protein-protein interactions as cataloged by [39], [40] and housed on the BAR server [54]. Protein-protein interaction confidence values were calculated as per [40] and clustering coefficients calculated as per [55]. mRNA transcript expression correlation scores were calculated as per [54] across approximately 1000 different microarray data sets from the AtGenExpress Consortium, comprising gene expression data from a developmental series [41], abiotic [56] and biotic stresses, and hormones and chemicals [57].

### Homology Relationships and Rendering of Sequence Data

Arabidopsis inparalogs and homologous sequences from *Populus trichocarpa* (poplar), *Medicago trunculata*, *Oryza sativa* (rice), and *Hordeum vulgare* (barley) were computed using OrthoMCL [58] by Rohan Patel and will be published elsewhere (Patel R and Provart NJ, manuscript in preparation). The sequence data comprising the homolog alignments are retrieved via webservices and aligned using MAFFT [59]. The model of the alignments and annotations are written using ActionScript and rendered with PaperVision3D.

### Mapping Single Nucleotide Polymorphism Data

Single nucleotide polymorphism data from [35], [36] were mapped to the coding and amino acid sequences displayed in the ePlant Sequence Explorer using Perl scripts and the BioPerl [51] library. The coding sequence of the query gene is aligned to the sequence fragments flanking and including polymorphic sites from [35], [36] using the (ends-free) Needleman-Wunsch dynamic programming algorithm and the FULLMAT substitution matrix implemented in EMBOSS. The FULLMAT matrix gives exact nucleotide/amino acid matches a score of 5 and mismatches a score of -4, which preserves the inequality “gap open < mismatch < gap extend < match”.

### Availability and Future Directions

The ePlant framework has the potential for many novel extensions to systems biology data integration. Careful statistical analyses of co-evolving amino acid sites that can reliably detect co-evolving protein sectors [60] and other metrics of evolutionary constraints [61] could be computed in high-throughput for entire proteomes and incorporated into the ePlant Sequence Explorer and Protein Structure Model Explorer. Genetic variation such as single nucleotide polymorphisms, e.g. from the 1001 Arabidopsis genomes project [62], could be similarly incorporated into the ePlant Sequence Explorer and Protein Structure Model Explorer. The ePlant Molecular Interactions Viewer could be extended by additional 3D layout algorithms and the inclusion of multipartite networks that include RNA, metabolic networks [27]–[29] and/or the small molecules of metabolism and signal transduction. These networks could incorporate data from biological small molecule resources such as the Golm Metabolome Database [63]. Computational modeling of many biological molecules in a complex cellular environment can provide invaluable insight into biological processes [64], [65]. Web-based 3D rendering engines can support libraries of complex physics functions for both objects and environments. This raises the possibility of computationally modeling the coordination between morphological development and molecular function using methods that are accessible to a broad range of researchers with minimal training in computer programming. Powerful server-side applications, such as the Bioconductor packages for R [66], E-Cell [67], [68], or Mathematica [53], could dynamically compute the properties of a biological model with user-provided parameters via web browsers and return these modeling data for rendering in a web browser. The anatomical and physiological descriptions of Arabidopsis currently used by ePlant are essentially cartoon representations. Ideally, the data display modules would render 3D representations of anatomy from direct measurements such as magnetic resonance imaging (MRI) and Z-stacks of confocal microscopic images. This has in principle been achieved by projects such as Cortona3D (web browser plug-in; http://www.cortona3d.com), which can render 3D objects reconstructed from MRI-based anatomical descriptions (Figure S3). Public repositories of 3D reconstructions of biological materials from MRI and microscopy studies already exist, such as the fMRI Data Center (http://www.fmridc.org) and the Cell Centered Database (http://ccdb.ucsd.edu).

The ePlant framework for systems biology analyses on the world wide web includes an open-source policy for community development, script-accessibility, functional independence of operating system type, and the ability to dynamically render object and environmental properties in 3D. The data display modules are designed to allow interaction with any other module which can accept and pass parameters through RESTful channels. This allows content creators to choose their tools, such as Processing (http://processing.org), for developing data display modules. In the current implementation of ePlant, 3D models are specified by Collada or PDB objects and the interaction with these objects is handled by a rendering engine through the web browser which incorporates biological data from web service streams and manages user input. RESTful web services allow flexible reformatting of the “raw” biological data using human-interpretable formats such as JSON. This enables the data to be efficiently served according to the specifications of any rendering engine. This is important, as technologies associated with 3D rendering on the web are advancing rapidly. Currently, the ePlant modules are rendered by either PaperVision3D/Flash, Google's O3D, or Jmol. When rendered using PaperVision3D (Figure A,C) the Collada models are loaded and manipulated quickly on a wide variety of computing systems tested. Google's O3D rendering engine (Figure B,D) generates much richer 3D depth and performance compared to the Flash rendering engine. However this plug-in version of O3D was experimental and has now been implemented using WebGL (http://www.khronos.org/webgl). WebGL is integrated with the HTML 5 “canvas” elements allowing declarative rendering of 3D content without the use of plug-in software (http://X3DOM.org) and is under active development to become the standard for 3D content on the web [69]. The fluidity of integration between the ePlant modules could be improved by implementing all of the modules with one rendering pipeline, such as WebGL. This would allow a seamless “zoom” from the function of organisms at the meter scale to the nanometer scale of protein function in one continuous environment. However, a combination of declarative and plug-in based 3D rendering will probably continue to be used. For example, it would require enormous programming efforts to replace Jmol's wealth of features for the study of molecules at the nanometer scale. It is also unlikely that the PDB format for the description of protein structures will change in the near future. We have demonstrated that protein structure models can be transcribed from the Protein Data Bank Markup Language (PDBML) specifications into the Collada mark-up language (Figure S4). However, the performance of these models when rendered using PaperVision3D/Flash is very poor compared to Jmol. Collada was designed as an intermediate exchange format for 3D content. Collada has the benefit of interchangeability with many common formats at the cost of relatively large file sizes with many unused properties. However, these Collada models can be easily parsed into more compact custom formats compatible with the evolving standards of 3D rendering engines.

ePlant is licensed under a Creative Commons Attribution-Share Alike 2.5 Canada License and can be freely accessed on the world wide web through standards-compliant web browsers at http://bar.utoronto.ca/eplant. The source code for the ePlant framework and the Collada models described in this article are available for download at http://3ddi.org and from SourceForge at http://sourceforge.net/projects/eplant/. The Phyre-predicted Arabidopsis protein structure models are available for download from the ePlant protein structure model explorer. The entire collection of protein structures is available upon request. All other Perl, CGI, Javascript, and ActionScript scripts are available upon request. Towards establishing community standards and developing ideas for the 3D display of biological data on the world wide web we have launched the 3D Data Display Initiative (http://3ddi.org). Technical topics concerning the ePlant source code and questions such as “which framework should be used for 3D rendering on the web?” or “which formats are suitable for describing 3D models of biological entities?” may be discussed.

## Supporting Information

Figure S1 Screenshot of the ePlant Protein Structure Explorer. A) The nucleotide binding site and B) the shikimate binding site described by CDD model cd00464 mapped onto a Phyre-predicted structure of an Arabidopsis shikimate kinase encoded by At2g21940. C) The crystallographic structure of At2g21940 (PDB:3NWJ) with the nucleotide binding site, as per [70], shown in yellow, D) The crystallographic structure of At2g21940 (PDB:3NWJ) with the shikimate binding site, as per [70], shown in yellow.
(TIF)

Figure S2 Screenshots from extant visualization tools for CDD models. A) Screenshot of a 2D schematic representations of CDD sites mapping to TBP1 encoded by At3g13445 returned from a BLAST analysis [71] (http://blast.ncbi.nlm.nih.gov). B) Screenshot of conserved domain structure for TBP1 encoded by At3g13445 returned from an InterProScan query [72] (http://www.ebi.ac.uk/Tools/InterProScan).
(TIF)

Figure S3 Screenshot of a 3D reconstruction of striatum and cerebral cortex of a monkey from the genus Callicebus from anti-KChIP2b immunostains. The reconstruction was rendered in a web browser using the Cortona3D plug-in (http://www.cortona3d.com). The 3D model was downloaded from the 3D Brain Objects (VRML) Database (http://brainmaps.org).
(TIF)

Figure S4 Rendering of structure models. Structure model of the Arabidopsis Leafy transcription factor bound to DNA (PDB accession 2VY2) transcribed from PDBML to the Collada format and rendered using the SwirlX3D viewer (http://www.pinecoast.com). The Leafy peptide bond alpha-carbon and nitrogen atoms are shown in green and blue, respectively. Atoms of the bound DNA molecule are shown in red.
(TIF)
