# Supplementary figures and images for: ePlant and the 3D Data Display Initiative: Integrative Systems Biology on the World Wide Web
Source: PLoS One. 2011 Jan 10;6(1):e15237. doi: 10.1371/journal.pone.0015237 (PMC3018417; doi:10.1371/journal.pone.0015237)

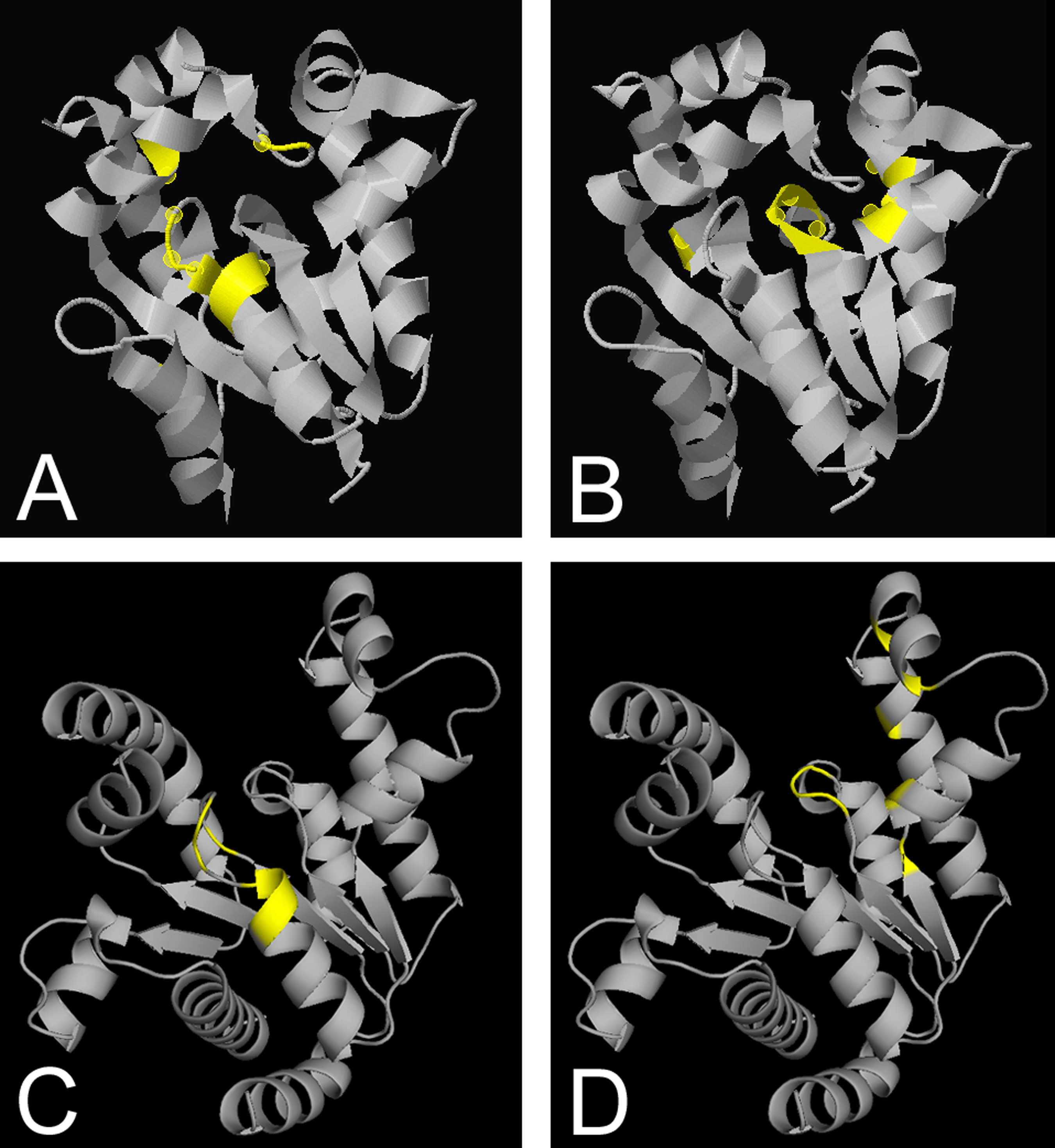

Supplement: Figure S1 — Screenshot of the ePlant Protein Structure Explorer. A) The nucleotide binding site and B) the shikimate binding site described by CDD model cd00464 mapped onto a Phyre-predicted structure of an Arabidopsis shikimate kinase encoded by At2g21940. C) The crystallographic structure of At2g21940 (PDB:3NWJ) with the nucleotide binding site, as per [70], shown in yellow, D) The crystallographic structure of At2g21940 (PDB:3NWJ) with the shikimate binding site, as per [70], shown in yellow. (TIF) [file pone.0015237.s001.tif]

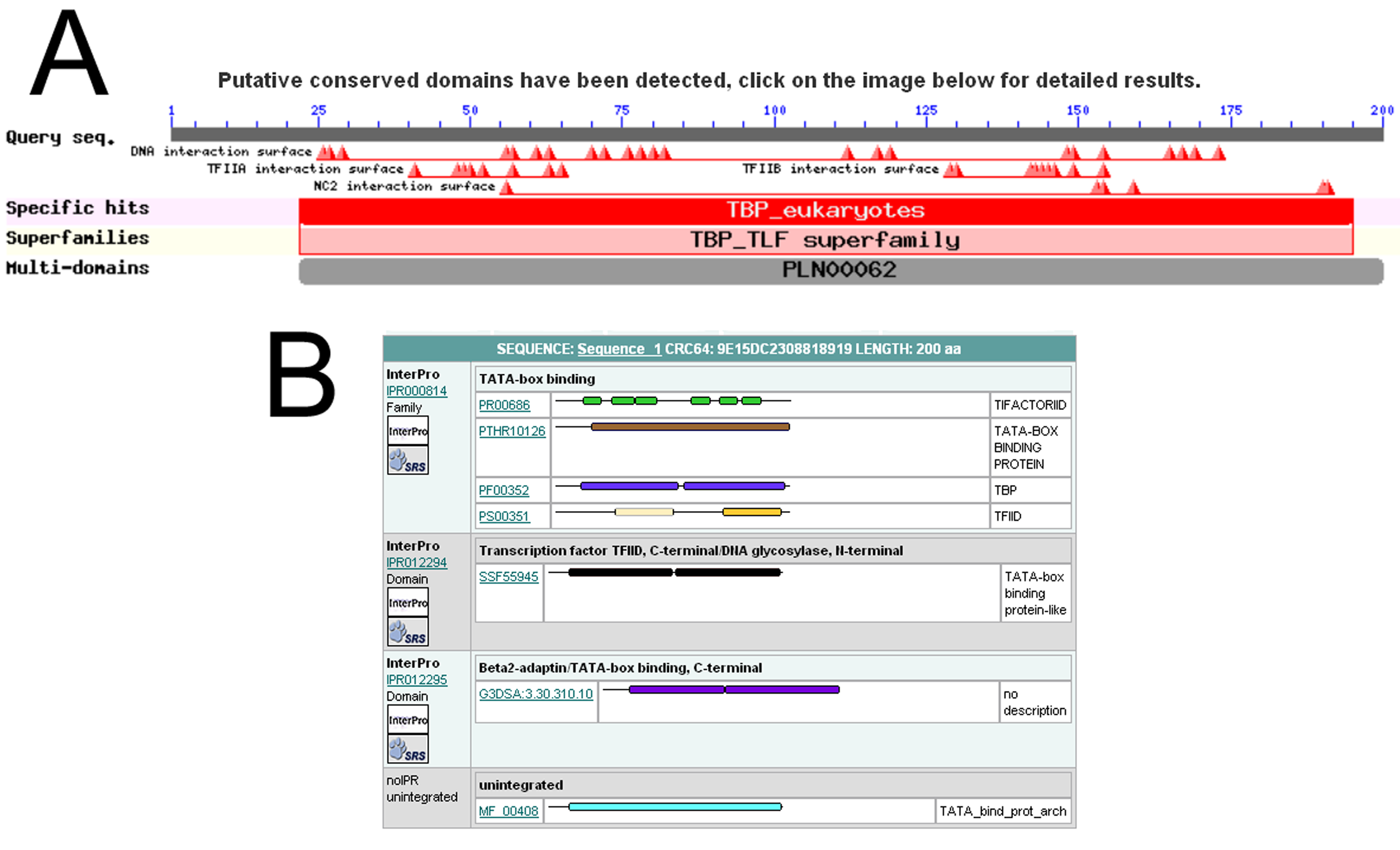

Supplement: Figure S2 — Screenshots from extant visualization tools for CDD models. A) Screenshot of a 2D schematic representations of CDD sites mapping to TBP1 encoded by At3g13445 returned from a BLAST analysis [71] (http://blast.ncbi.nlm.nih.gov). B) Screenshot of conserved domain structure for TBP1 encoded by At3g13445 returned from an InterProScan query [72] (http://www.ebi.ac.uk/Tools/InterProScan). (TIF) [file pone.0015237.s002.tif]

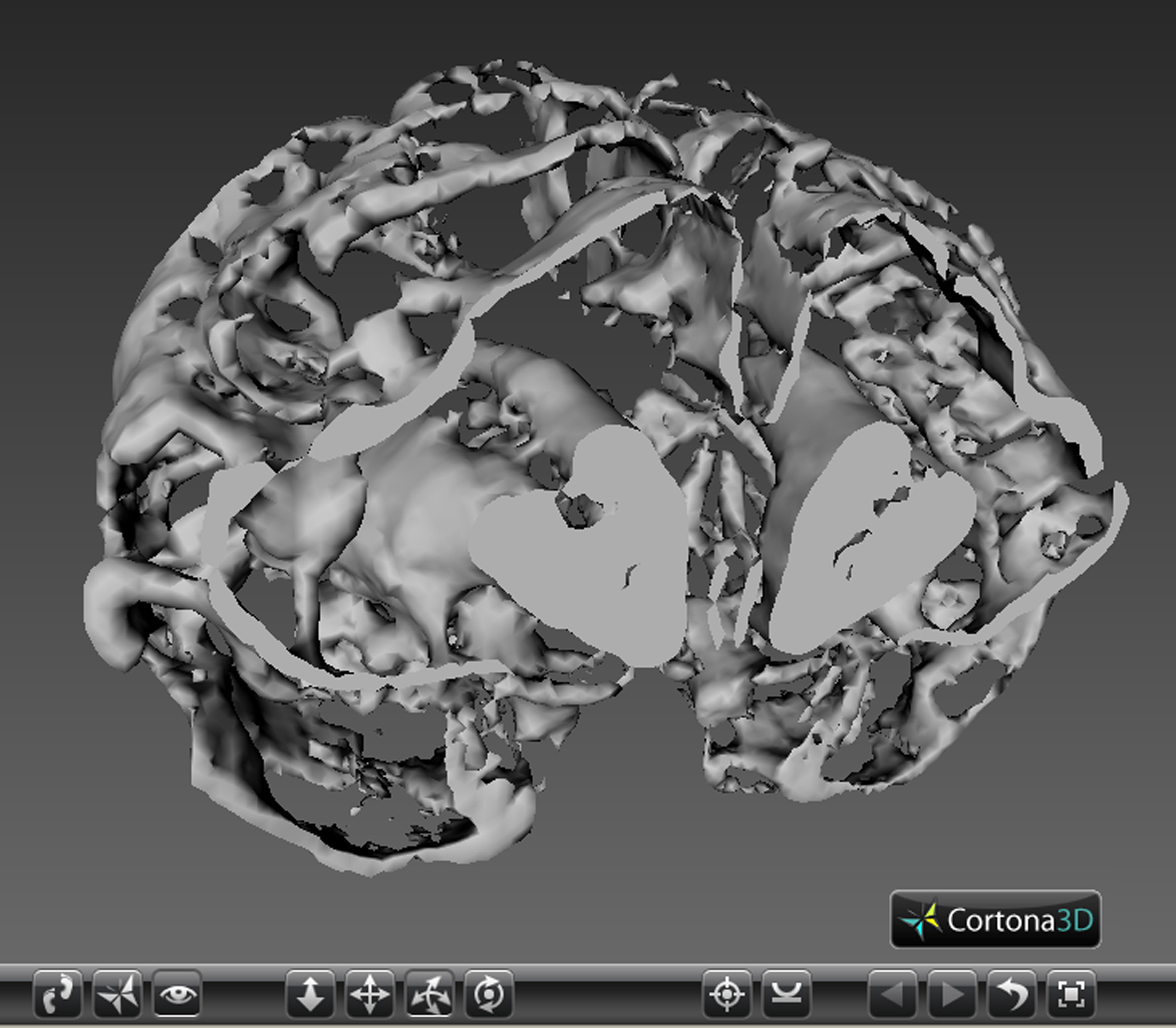

Supplement: Figure S3 — Screenshot of a 3D reconstruction of striatum and cerebral cortex of a monkey from the genus Callicebus from anti-KChIP2b immunostains. The reconstruction was rendered in a web browser using the Cortona3D plug-in (http://www.cortona3d.com). The 3D model was downloaded from the 3D Brain Objects (VRML) Database (http://brainmaps.org). (TIF) [file pone.0015237.s003.tif]

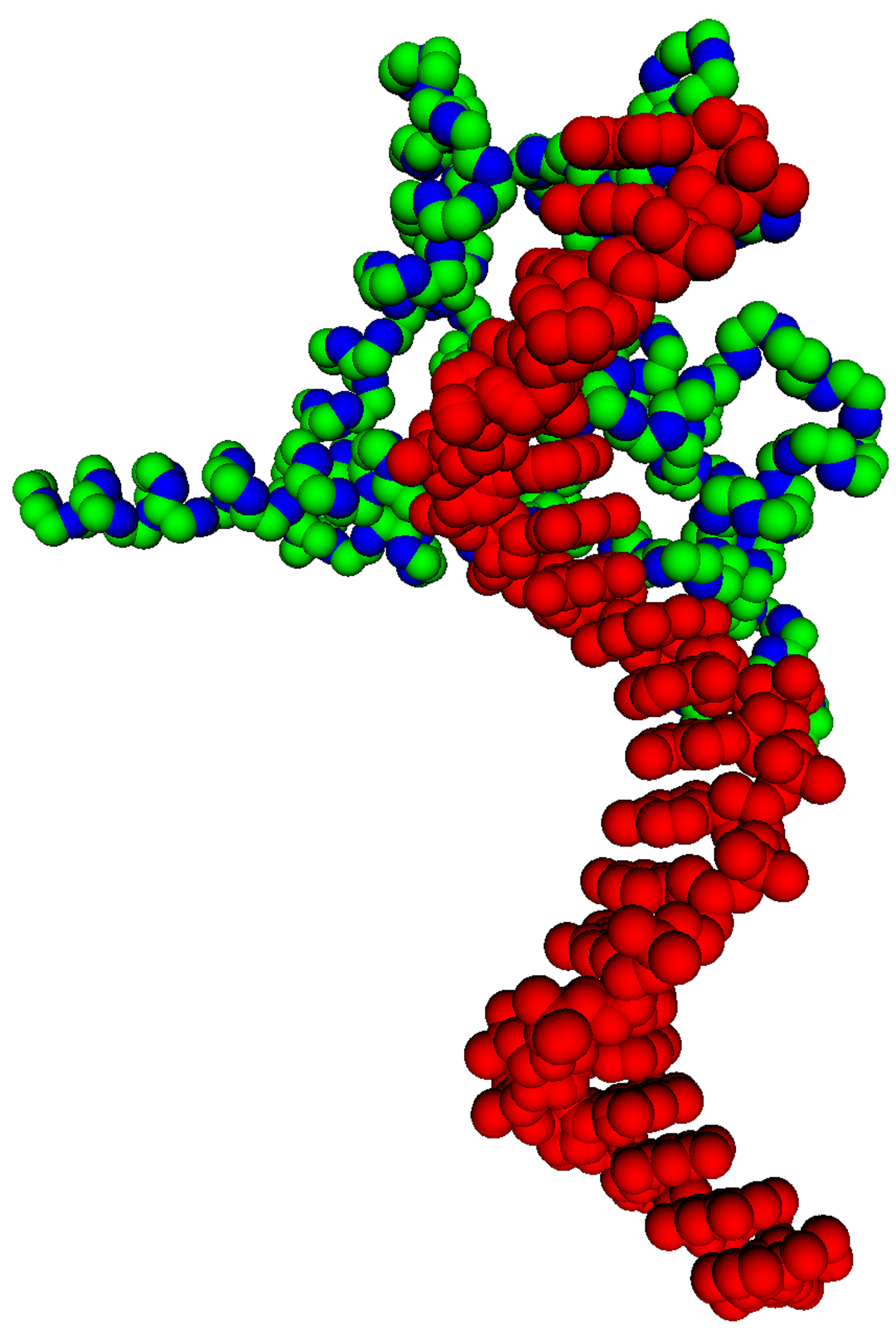

Supplement: Figure S4 — Rendering of structure models. Structure model of the Arabidopsis Leafy transcription factor bound to DNA (PDB accession 2VY2) transcribed from PDBML to the Collada format and rendered using the SwirlX3D viewer (http://www.pinecoast.com). The Leafy peptide bond alpha-carbon and nitrogen atoms are shown in green and blue, respectively. Atoms of the bound DNA molecule are shown in red. (TIF) [file pone.0015237.s004.tif]
